# Gut microbiota-regulated glutathione metabolic rhythms restore obesity-induced colonic inflammatory oscillations

**DOI:** 10.1080/19490976.2026.2670048

**Published:** 2026-05-09

**Authors:** Zhenting Zhao, Renjie Shi, Jin Ye, Danna Wang, Beita Zhao, Bo Ren, Luanfeng Wang, Xiaoning Liu, Xuebo Liu

**Affiliations:** a College of Food Science and Engineering, Northwest A&F University, Yangling, China; b School of Food Science and Pharmaceutical Engineering, Nanjing Normal University, Nanjing, China; c College of Food Science and Engineering, Nanjing University of Finance and Economics/Collaborative Innovation Center for Modern Grain Circulation and Safety, Nanjing, China; d Institute of Molecular and Cell Biology, Agency for Science, Technology and Research, Proteos, Singapore

**Keywords:** Metabolic syndrome, High-fat diet, Gut microbiota, Inflammatory oscillations, Glutathione metabolism, Circadian rhythm, Fructo-oligofructose

## Abstract

Obesity disrupts circadian inflammatory rhythms, a defining feature of metabolic syndrome. However, the mechanisms connecting microbial and host circadian communication remain unclear. By using the fermentable fiber fructo-oligosaccharide (FOS) to restore microbial rhythmicity, we found that a high-fat diet (HFD) disrupts microbiota-regulated oscillations in glutathione metabolism, thereby dampening colonic inflammatory rhythms independently of the core clock machinery. Fecal microbiota transplantation (FMT) further supported a causal role for rhythmic fecal microbial signals in restoring inflammatory oscillations. Integrated multi-omics analysis highlighted circadian glutathione metabolism as a prominent candidate pathway linking microbial rhythmicity to host inflammatory oscillations. Importantly, colon-specific knockdown of *Gclc*, the rate-limiting enzyme in glutathione synthesis, abolished the restorative effects of microbial rhythms, functionally positioning host glutathione metabolism as a critical downstream mediator. Collectively, our study supports the existence of a microbiota-glutathione axis that contributes to the regulation of colonic inflammatory rhythms, uncovering a new chronobiological layer of microbial control over host inflammation.

## Graphical abstract

**Figure:**
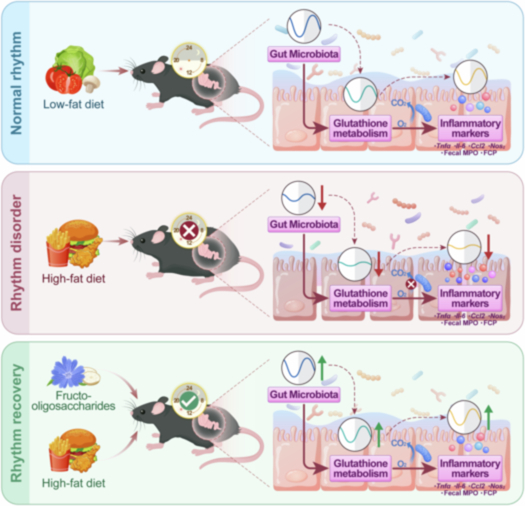

## 1. Introduction

Circadian rhythms represent a fundamental adaptive mechanism, enabling organisms to anticipate and respond to daily environmental fluctuations.^1^^,^^2^ Within the gastrointestinal tract, these rhythms are paramount, governing critical processes ranging from epithelial renewal to immune surveillance and the resolution of inflammatory responses.^3-5^ The precise temporal orchestration of these events is essential for maintaining intestinal homeostasis, and its breakdown is a hallmark of various pathological states.^6-8^ This is exemplified in metabolic syndrome, a cluster of conditions that includes obesity, insulin resistance, and hypertension.^9^^,^^10^ Prolonged high-fat diet (HFD) feeding profoundly disrupts circadian inflammatory rhythms, establishing a robust model for their dysregulation.^11^ This disruption is characterized by both elevated inflammatory tone and a marked loss of rhythmic precision, fostering chronic, non-resolving inflammation that contributes to disease pathogenesis.^12^ While the host's intrinsic circadian clock, which is governed by core transcription-translation feedback loops, is an established regulator, it represents only one part of a complex equation. A burgeoning body of evidence now underscores the gut microbiota, a dynamic and metabolically active ecosystem that exhibits its own intrinsic diurnal fluctuations, as a pivotal non-cell-autonomous orchestrator of host physiology.^13-15^ However, the precise molecular mechanisms that synchronize microbial rhythmicity with host inflammatory oscillations remain a significant and unresolved question in biology.

The composition and functional activity of the gut microbiota are dynamic, displaying robust and predictable diurnal oscillations shaped by host diet and circadian signals.^16^ Importantly, microbial rhythmicity is not a passive byproduct but plays an active role in regulating host circadian functions.^17^ Evidence demonstrates a causal link between microbiota temporal dynamics and inflammatory rhythms. For example, daily oscillations of commensals such as *Parabacteroides distasonis* modulate the cyclical pattern of arthritic inflammation,^18^ whereas resynchronization of rhythmic taxa including *Lactobacillus* and *Lachnoclostridium* is linked to improvements in non-alcoholic fatty liver disease.^19^ Conversely, environmental stressors such as HFD feeding markedly disrupt microbial oscillations, causing loss of rhythmicity in inflammation-associated taxa and fostering arrhythmic, low-grade inflammation characterized by elevated pro-inflammatory cytokines and impaired barrier integrity.^20^ Collectively, these findings establish the gut microbiota as a central mediator translating dietary and temporal cues into host inflammatory responses. However, the signaling molecules and pathways underlying microbiota-host chrono-communication remain poorly defined, highlighting a critical knowledge gap.

To move beyond correlation and define the causal mechanisms through which microbial rhythms influence host inflammatory cycles, we sought to experimentally restore a healthy rhythmic community in a dysbiotic context. We reasoned that such an intervention could simultaneously correct microbial ecological imbalance and temporal disorder, thereby providing a powerful strategy to identify the signaling pathways synchronized by rhythmic microbiota and relayed to the host. We applied this strategy by leveraging the properties of fermentable dietary fibers, which are potent modulators of gut microbial composition and function.^21^^,^^22^

We employed fructo-oligofructose (FOS), a well-characterized fermentable fiber, to reconstitute microbial rhythmicity in mice with HFD-induced obesity, a model characterized by profound disruption of both microbial and inflammatory oscillations.^23-25^ We postulated that targeted restoration of microbial rhythms would be sufficient to reinstate colonic inflammatory oscillations. Furthermore, by integrating time-resolved metabolomic profiling of colonic contents with transcriptomic analysis of colonic tissues, we aimed to pinpoint the metabolic pathways entrained by microbial rhythms that transmit these signals to the host epithelium.

Our multi-omics analyzes identified glutathione metabolism as a central pathway mediating microbiota-host cross-talk. Glutathione, the cell’s primary antioxidant, is essential for redox homeostasis and the modulation of inflammatory signaling.^26^^,^^27^ Beyond its canonical role, emerging evidence indicates that glutathione levels and the activity of its rate-limiting enzyme, glutamate-cysteine ligase (GCL),^28^ whose catalytic subunit is encoded by *Gclc*, exhibit diurnal oscillations in metabolic tissues such as the liver and intestine,^29^ suggesting circadian regulation.^30^^,^^31^ While this regulation has traditionally been ascribed to host clock genes,^32^ it remained unclear whether and how microbial rhythms directly govern the circadian dynamics of colonic glutathione metabolism to pace inflammatory oscillations. In this study, we integrate chronobiological, microbiological, genetic, and multi-omics approaches to address this question. Our findings establish a critical microbiota-glutathione metabolic axis indispensable for maintaining circadian inflammatory rhythms. This discovery not only extends the role of glutathione beyond redox balance but also reveals a fundamental chronobiological mechanism through which the rhythmic gut ecosystem orchestrates host inflammatory homeostasis, providing new mechanistic insights into the interplay among diet, microbiota, and host health.

## 2. Materials and methods

### 2.1. Mice

Male C57BL/6J mice (6−8 weeks old), maintained under specific pathogen-free (SPF) conditions, were procured from Beijing HFK Bioscience Co., Ltd. Additionally, Bmal1-FLOX and Bmal1-IKO mice (C57BL/6J background) were sourced from GemPharmatech Co., Ltd. All animals were housed in a controlled environment with a 12-hour light/dark cycle (lights on at 08:00, designated as ZT0). After a two-week acclimatization period, animals were divided into three dietary groups (Table S8): Low-fat diet (LFD), high-fat diet (HFD; 60% kcal from fat), and high-fat diet supplemented with 10% fructo-oligosaccharides (HFD-FOS). After seven weeks of intervention, euthanasia was performed using isoflurane anesthesia and cervical dislocation at six circadian time points: ZT0 (08:00), ZT4 (12:00), ZT8 (16:00), ZT12 (20:00), ZT16 (00:00), and ZT20 (04:00).

Experimental units were individual mice. Across all experimental conditions, each experimental group contained *n* = 6 mice per sampling time point. Depending on the specific experiment, mice were sampled at either multiple circadian time points or selected time points relevant to the study design. In total, 360 mice were used across all experiments. Group allocations and sampling schemes are provided in the figure legends. No animals were excluded unless they met predefined humane endpoint criteria.

Randomization, blinding, and confounder control. Mice were randomly assigned to groups after acclimatization using a random number generator. Cage placement, handling sequence, and sampling order were rotated systematically to minimize cage effects and time-of-day handling bias. Group allocation was known to personnel responsible for animal assignment and routine husbandry; however, standardized procedures were followed for sample collection without reference to group labels. Investigators responsible for outcome assessment and data analysis were blinded to group allocation until completion of primary analyzes.

Sample size justification. The sample size was based on our previous work and published studies employing comparable diet-induced obesity and circadian rhythm mouse models, in which *n* = 6 animals per group per time point reliably provides sufficient power to detect biologically meaningful metabolic and circadian differences while accounting for inter-individual and rhythm-related variability.

### 2.2. Gut microbiota depletion through antibiotic treatment

To verify microbiota dependence, an antibiotic mixture was added to the drinking water to deplete the gut microbiota (ampicillin 0.2 g/L, neomycin 0.2 g/L, metronidazole 0.2 g/L, and vancomycin 0.1 g/L). Mice received antibiotics continuously for 2 weeks, followed by a maintenance regimen of 4 d on antibiotics and 3 d on sterile water each week until the end of the experiment.

To validate microbiota rhythmicity, recipient mice were pretreated with a standard broad-spectrum antibiotic cocktail in the drinking water for 14 consecutive days (vancomycin 0.5 g/L, ampicillin 1 g/L, neomycin 1 g/L, and metronidazole 1 g/L). Antibiotic water was protected from light and replaced every 1-2 d. Mice were then switched to sterile, antibiotic-free water for 1-2 d (washout/rest) before initiating fecal microbiota transplantation.

Microbiota depletion in the corresponding antibiotic-treated groups was validated by plating fecal suspensions on BHI agar under both aerobic and anaerobic conditions. Representative plate images are provided in the supplementary materials (Fig S1).

### 2.3. Fecal microbiota transplantation (FMT)

To validate microbiota rhythmicity, the gut microbiota was first depleted by antibiotic treatment. Then, 100 mg of fecal pellets were collected daily at ZT0 and ZT12 from donor mice in the HFD-FOS group for two weeks. After collection, fresh feces were suspended in 1 mL of sterile PBS and homogenized thoroughly. The suspension was then centrifuged at 400 × g for 10 minutes to remove large particulate debris, and 200 μL of the prepared fecal suspension was administered daily to each recipient mouse by oral gavage while maintained on HFD.

### 2.4. AAV adenovirus construct

The purpose of this system is to introduce dual-sgRNA-directed genomic cleavage to knock down the expression of the target gene.

(1) Vector Construction: The vector rAAV-EF1a-Cas12-dual-sgRNA was digested with BbsI, and the U6-scaffold-sgRNA1/sgRNA2 fragment was synthesized. A Cas12f-based dual-sgRNA knockout AAV vector was constructed using the Golden Gate assembly method.
(2) Plasmid Preparation and Viral Packaging: Endotoxin-free plasmid DNA was extracted, followed by AAV viral packaging.
(3) AAV Packaging in HEK293 Cells: Viral packaging was performed in HEK293 cells using a three-plasmid system, including AAV-Helper, RC8 (serotype 8), and the constructed rAAV-EF1a-Cas12-dual-sgRNA vector. Cells cultured in 150 cm^2^ dishes were harvested at 48 h and 96 h post-transfection, and the supernatants were collected. Viral particles were purified and concentrated via ultracentrifugation and ultrafiltration. The viral titer and genome copy number were quantified using qPCR, and animal injections were performed based on the calculated titers.

### 2.5. AAV adenovirus enema

(1) Mice were fasted overnight, and 20 mM *N*-acetyl-L-cysteine (NAC) was used to facilitate mucus extrusion from intestinal crypts.
(2) Pilocarpine was administered intraperitoneally at a dose of 30 mg/kg at least 45 minutes before the procedure.
(3) Mice were anesthetized with isoflurane.
(4) The colon was washed for approximately 30 minutes via rectal injection of 300 μL of 20 mM NAC using a 1 mL syringe fitted with a 2.5 cm straight stainless steel needle.
(5) A polyethylene tube was attached to the tip of a 1 mL syringe and inserted 4 cm into the mouse colon from the anus. Then, 200 μL of AAV solution was slowly administered via enema.
(6) Following injection, mice were held in a vertical inverted position for at least 1 minute to ensure even distribution of the viral solution within the colon.
(7) After recovery from anesthesia, mice were allowed to resume normal food and water intake.

### 2.6. 16S rRNA amplicon sequencing

Nucleic acids were extracted using the TGuide S96 Magnetic Bead Fecal Genomic DNA Extraction Kit. The V3-V4 region of the bacterial 16S rRNA gene was amplified using the following primers: forward 5'-ACTCCTACGGGGAGGCAGCA-3', reverse 5'-GGACTACHVGGGTWTCTAAT-3'. PCR products were analyzed using the LabChip GX Touch system (PerkinElmer, model CLS137031/E). Following amplification, sequencing libraries were constructed, and samples were sequenced on the Illumina NovaSeq 6000 platform.

### 2.7. Metabolomics analysis

A 50 mg sample was accurately weighed, freeze-milled, and extracted at low temperature using an appropriate solvent. After centrifugation, the supernatant containing metabolites was collected and analyzed by LC-MS using an ultra-high-performance liquid chromatography tandem Fourier transform mass spectrometry system (UHPLC-Q Exactive HF-X, Thermo Fisher).Raw data were imported into Progenesis QI software (Waters Corporation, Milford, USA) for baseline correction, peak detection, integration, retention time correction, and alignment, resulting in a final data matrix containing retention time, mass-to-charge ratio (m/z), and peak intensity.Metabolite identification was performed by matching MS and MS/MS spectral data to entries in metabolomic databases, with a mass error threshold of less than 10 ppm. Metabolites were identified based on secondary spectral matching scores. Major databases used included the Human Metabolome Database (HMDB, http://www.hmdb.ca/) and METLIN (https://metlin.scripps.edu/), along with other public metabolomics databases.

### 2.8. RNA sequencing analysis

Total RNA was extracted from mouse colon tissue using TRIzol reagent, followed by purification with an RNA Purification Kit. RNA integrity was assessed using Biowest agarose gel electrophoresis. The SMART-Seq v4 Ultra Low Input RNA Kit (micro-volume method) was used for cDNA synthesis.Sequencing libraries were constructed using the SMART-Seq v4 Ultra Low Input RNA Kit (micro-volume method), and sequencing was performed on a NovaSeq X Plus platform using the NovaSeq Reagent Kit.

### 2.9. qRT-PCR

Total RNA was extracted from mouse colonic tissues using TRIzol reagent, and reverse transcription was carried out using the All-in-One Step RT Mixture (Beijing Allmeek Biotechnology Co., Ltd.). Relative mRNA expression levels were quantified by real-time PCR using a QuantStudio 5 system (Applied Biosystems, Singapore), and data were analyzed using the 2^-ΔΔCt^ method. The gene-specific primers used in this study are listed in Table S6.

### 2.10. H&E stain

Colonic tissues were fixed, embedded in paraffin, and sectioned at a thickness of 5 μm. The sections were stained with hematoxylin and eosin (H&E), and images were captured using an IX73 inverted fluorescence microscope (Olympus, Japan). Histological scoring of the tissue sections was performed as described in Table S7.

### 2.11. Alcian blue staining

Colonic tissues were embedded in paraffin, sectioned at 5 μm thickness, and stained with Alcian blue. Images were captured using an IX73 inverted fluorescence microscope (Olympus, Japan). Positive cells were identified based on their comet-like morphology, characterized by intense blue staining at the apical side of the colonic crypts within the selected frame.

### 2.12. Hypoxia staining

Hypoxia was detected by intraperitoneal injection of pimonidazole hydrochloride (60 mg/kg; Hypoxyprobe™-1 Kit, Hypoxyprobe) one hour prior to euthanasia. Colonic tissues were fixed in 10% phosphate-buffered formalin, paraffin-embedded, and sectioned. Immunostaining for pimonidazole adducts was performed using FITC-conjugated anti-pimonidazole mouse monoclonal antibodies. Sections were scored based on the degree of colonic epithelial hypoxia as follows: 0 = no hypoxia, 1 = mild focal hypoxia, 2 = moderate multifocal hypoxia, and 3 = severe diffuse hypoxia. Representative images were acquired using an IX73 inverted fluorescence microscope (Olympus, Japan).

### 2.13. FCP and MPO measurements

Mouse fecal samples were homogenized in 1 mL of sterile PBS and centrifuged at 3000 × g for 5 minutes. The supernatant was collected for quantification of fecal calprotectin (FCP) levels using the Mouse S100A8/S100A9 Heterodimer ELIZA Kit (R&D Systems, USA), and fecal myeloperoxidase (MPO) levels were measured using the Mouse MPO ELIZA Kit (BioVision, Milpitas, CA) according to the manufacturers’ instructions.

### 2.14. ATP measurements

Colonic tissues were collected and deproteinized using a Deproteinization Sample Preparation Kit (BioVision, Milpitas, CA) according to the manufacturer's instructions. ATP levels in colonic tissue lysates were measured using an ATP Colorimetric Assay Kit (BioVision, Milpitas, CA), following the manufacturer's protocol.

### 2.15. PDH activity measurements

To assess pyruvate dehydrogenase (PDH) activity, primary colon cells were isolated as previously described, lysed in RIPA buffer, and centrifuged at 13,000 rpm for 5 minutes at room temperature to remove cellular debris. PDH activity in the supernatant was quantified using a PDH Activity Assay Kit (BioVision, Milpitas, CA) according to the manufacturer’s protocol.

### 2.16. Lactate measurements

Colonic tissue was collected and deproteinized using a deproteinization sample preparation kit (Biovision, Milpitas, CA), following the manufacturer’s instructions. Lactate levels in colonic lysates were measured using the Lactate Colorimetric Assay Kit II (Biovision, Milpitas, CA), following the manufacturer’s instructions.

### 2.17. GSH, GSSG, and MDA measurements

Colonic tissues were collected and processed according to the manufacturers’ instructions. GSH, GSSG, and MDA levels in colonic tissue lysates were measured using corresponding assay kits (Abcam, Cambridge, UK) following the manufacturers’ protocols. The GSH/GSSG ratio was calculated accordingly.

### 2.18. Integrated analysis of the transcriptome and metabolome

Transcriptomic and metabolomic data were analyzed using the Majorbio Cloud Platform (https://cloud.majorbio.com/).

### 2.19. Statistical Analysis

The primary outcome measure used for sample-size justification was the diurnal variation and rhythmic oscillations in colonic inflammatory cytokine expression (*Tnfα*, *Il6*, *Ccl2*, *Nos2*). Secondary outcome measures included colonic histopathology scores, goblet cell counts, fecal inflammatory markers (MPO, FCP), tissue metabolic measurements (ATP, PDH, lactate), mucosal hypoxia, and multi-omics endpoints (transcriptomics and metabolomics). All outcome measures and quantification methods are described in the corresponding figure legends and Supplementary Methods. Statistical analyzes were conducted using GraphPad Prism software (version 9.4.1), with quantitative results expressed as mean ± SD unless specified for 16S rRNA amplicon sequencing, metabolomic, or transcriptomic datasets. The Wilcoxon test and two-tailed unpaired Welch’s t-test were used to compare two groups. One-way ANOVA followed by Tukey’s post hoc test was used to compare three or more groups. If heteroscedasticity was detected (Brown-Forsythe test, *p* < 0.05), Welch’s ANOVA followed by Dunnett’s test was applied instead of ordinary ANOVA. Statistical significance thresholds were defined as follows: * indicating *p* < 0.05, ** corresponding *p* < 0.01, *** representing *p* < 0.001, and **** denoting *p* < 0.0001, with these criteria applied uniformly to both parametric t-tests and ANOVA-derived multiple comparisons. Circadian rhythm patterns were assessed through computational analysis with the JTK_CYCLE algorithm (MetaCycle package, version 1.2.0) implemented in the R statistical programming environment. The circle plots were drawn using ChiPlot (https://www.chiplot.online/).

### 2.20. Patient and Public Involvement

Patients or members of the public were not involved in the design, conduct, reporting, or dissemination plans of this research.

## 3. Results

### 3.1. Obesity disrupts diurnal inflammatory variations restored by microbiota-targeted interventions

**Figure 1.**
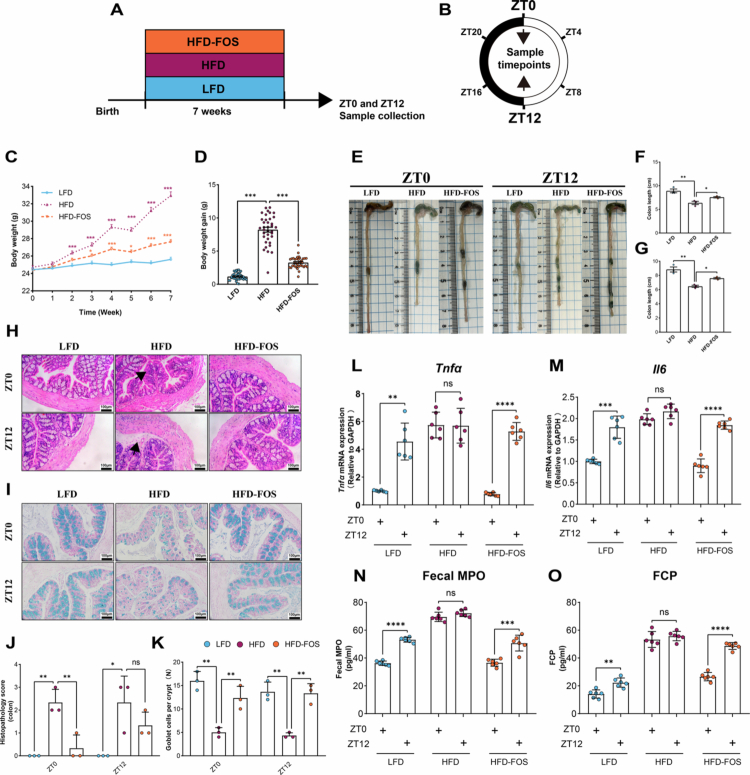
Obesity disrupts diurnal inflammatory variations restored by microbiota-targeted interventions. **A-B** Schematic of the experimental design: (A) Experimental design, (B) Detection time points. **C-D** Weight Change Chart: (C) Weight Change, (D) Weight Gain. **E-I** Representative colonic histomorphology and mucosal status at ZT0 and ZT12 in mice on different diets (*n* = 3/group/time point). **E** Colon length. **F** Difference in colon length at ZT0. **G** Difference in colon length at ZT12. **H** Colonic H&E staining. **I** Colonic Alcian blue staining. **J** Pathological scoring of colonic H&E staining. **K** Goblet cell count per colonic crypt across dietary groups. **L-O** Diurnal variations in colonic inflammatory markers in mice on different diets (*n* = 6/group/time point): (L) *Tnfα*, (M) *Il6*, (*N*) Fecal MPO, (O) Fecal FCP. One-way ANOVA was used to calculate *p* values in (C)-(D), (F)-(G) and (J)-(K). NS, *p* > 0.05, *p < 0.05, **p < 0.01, ***p < 0.001. Two-tailed unpaired Welch’s t test was used to calculate *p* values in (L)-(O). NS, *p* > 0.05, **p < 0.01, ***p < 0.001, ****p < 0.0001.

After confirming successful establishment of the HFD-induced obesity phenotype by body-weight and related metabolic measurements (Figure 1A-D), including weekly food intake and food efficiency ratio (Fig S2), we first assessed the histomorphological changes and mucus barrier function of the colon at two representative circadian phases, ZT0 (08:00) and ZT12 (20:00), in mice subjected to various dietary interventions. The results showed that the colonic mucosa was damaged at both ZT0 and ZT12 following HFD feeding, as evidenced by shortened colon length, mild leukocyte infiltration, submucosal edema, and depletion of mucin-producing goblet cells in the colonic epithelium. These pathological changes were alleviated in the HFD-FOS group (Figure 1E-K; Fig S3). Subsequently, we assessed the levels of inflammatory markers and their differences in murine colonic tissues under different dietary interventions at ZT0 and ZT12. The results indicated that *Tnfα*, *Il6*, fecal myeloperoxidase (MPO), and calprotectin (FCP) were markedly upregulated in the colonic mucosa of HFD-fed mice. FOS intervention reduced these inflammatory markers (Fig S4). Notably, diurnal variations in colonic pro-inflammatory cytokines *Tnfα* and *Il6* were observed in the LFD and HFD-FOS groups. In contrast, HFD-fed mice maintained elevated levels of *Tnfα* and *Il6* across diurnal phases without significant variation (Figure 1L-M). The trends of fecal MPO and FCP were consistent with those of the pro-inflammatory cytokines (Figure 1N-O). These findings indicate that FOS ameliorates chronic low-grade inflammation and restores diurnal variations in inflammatory markers in the colon of HFD-fed mice.

### 3.2. Gut microbiota underpin diurnal variation in colonic inflammation

Given the influence of the circadian system on metabolic rhythms, we first investigated whether circadian clock genes contribute to the modulation of diurnal variations in colonic inflammatory markers. Notably, we found that FOS restored the diurnal variations of colonic inflammatory markers (*Tnfα*, *Il6*, MPO, and FCP) in HFD-fed mice, including in Bmal1-intestine-specific knockout (Bmal1-IKO) mice (Figs S5A-G). This suggests that circadian clock genes are not key mediators of the modulation of diurnal variations in colonic inflammatory markers by FOS. Next, considering the role of dietary components in regulating gut microbiota structure, we administered antibiotics in the drinking water of mice to deplete the gut microbiota. Notably, the restorative effect of FOS on diurnal variations in colonic inflammation was absent in HFD-fed mice (*p* > 0.05, Figs S5H-N). The findings demonstrate that FOS modulates diurnal variations in colonic inflammatory markers via the gut microbiota, rather than through host circadian clock genes.

**Figure 2.**
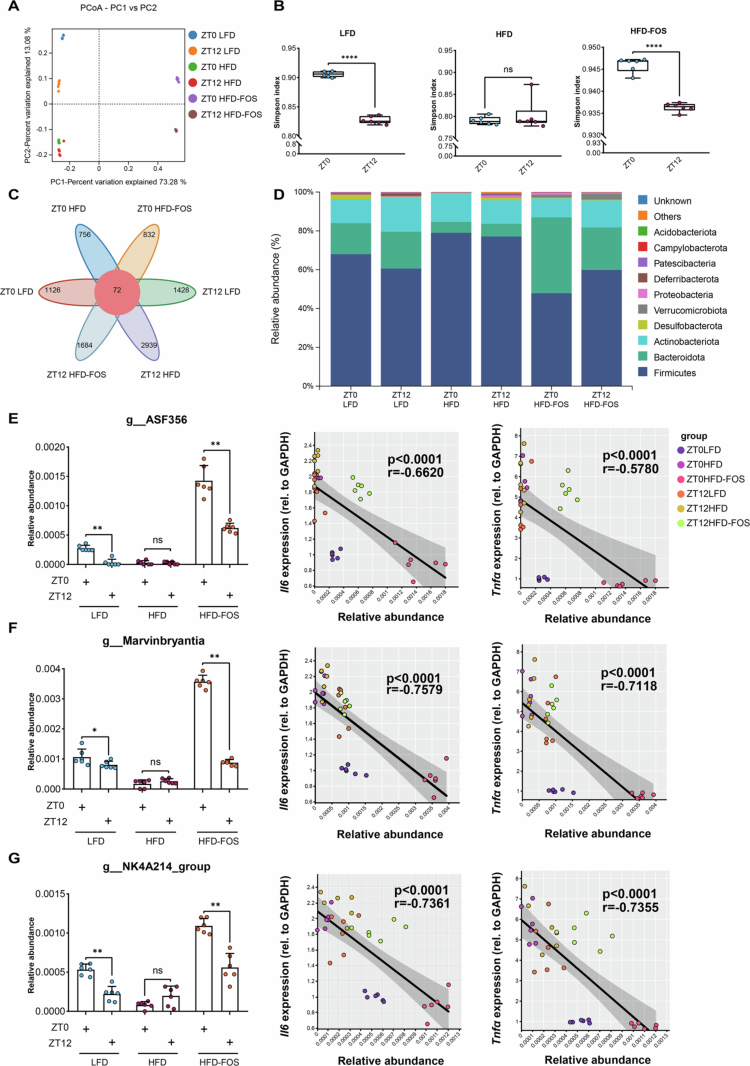
Gut microbiota underpin diurnal variation in colonic inflammation. **A** Comparison of *β*-diversity based on Bray-Curtis distance between groups. **B** Comparison of the *α*-diversity index between groups. **C** Venn diagrams comparing groups. **D** Bar graph of relative abundance of phylum-level microbiota between groups. **E-G** Diurnal relative abundance of representative genera with significant variations only in the LFD and HFD-FOS groups, but not in the HFD group, and their correlation with pro-inflammatory cytokines *Il6* and *Tnfα*: (E) *ASF356*, (F) *Marvinbryantia*, (G) *NK4A214_group* (*n* = 6/group/time point). Wilcoxon test was used to calculate *p* values in (E)-(G). NS, *p* > 0.05, *p < 0.05, **p < 0.01.

To delineate the microbial drivers of diurnal inflammatory modulation in response to dietary and temporal cues, we first analyzed the gut microbiota composition in three groups of mice at ZT0 and ZT12. We observed that both diet composition and time influenced gut microbiota composition (Fig S6; Figure 2A and C). Notably, diurnal microbiota diversity in the HFD group did not show significant differences in *β*-diversity (via principal coordinate analysis, PCoA) or *α*-diversity (via the Simpson index) (*p* > 0.05, Figure 2A-B), suggesting that lipid-specialized taxa may displace temporally regulated commensals through niche competition, thereby establishing an alternative equilibrium state. We then assessed the taxonomic composition of the colonic microbiota. Dietary composition analysis revealed a marked elevation of Firmicutes, Cloacimonadota, and Acidobacteriota, along with a substantial depletion of Patescibacteria and Deferribacteria in HFD group vs. LFD group (Fig S6B). FOS supplementation increased the intestinal abundance of Bacteroidota, Verrucomicrobiota, Cyanobacteria, and Deferribacterota, while decreasing the abundance of Gemmatimonadota, Myxococcota, Patescibacteria, Desulfobacterota, and Firmicutes under high-fat dietary conditions (Fig S6C). Temporally, the abundance of Firmicutes, Actinobacteriota, and Verrucomicrobiota showed elevated nocturnal levels in the HFD-FOS group, while Cyanobacteria, Proteobacteria, and Bacteroidota were more abundant during the day (Fig S6I). Notably, Firmicutes exhibited significant diurnal differences in the LFD and HFD-FOS groups, whereas these differences disappeared in the HFD group (Figs S6G-I). At the genus level, differences in microbiota composition were also observed over time and in response to dietary changes (Figs S6J-M). Notably, genera *ASF356*, *Marvinbryantia*, and *NK4A214_group* were highly abundant and exhibited significant diurnal oscillations in the HFD-FOS and LFD groups. In contrast, their abundance was low and diurnal oscillations were absent in the HFD group. Moreover, all of these genera showed a significant negative correlation with inflammatory markers (Figure 2E-G), consistent with previous reports.^33-35^ These findings suggest that the HFD disrupted the structure of the gut microbiota and altered the diurnal pattern of inflammation-associated microbiota, an effect reversed by FOS supplementation.

### 3.3. FOS reshapes microbial ecology by promoting rhythmically oscillating anti-inflammatory taxa

**Figure 3.**
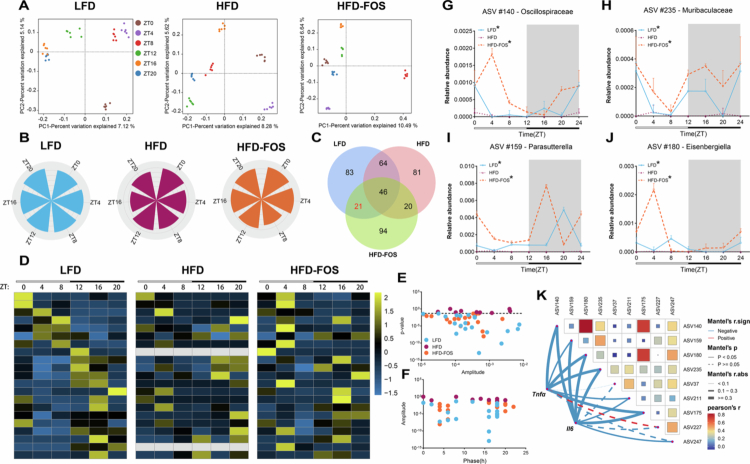
FOS reshapes microbial ecology by promoting rhythmically oscillating anti-inflammatory taxa. **A** Comparison of *β*-diversity based on Bray-Curtis distance (*n* = 6/group/time point). **B** Top-phase plots of total microbiota reads at six time points. **C** Screening of rhythmically oscillating genera in different groups by JTK_CYCLE algorithm and Venn diagrams to reflect the intersection of oscillating genera between groups. **D-F** 21 ASVs showing rhythmic oscillations in both the LFD and HFD-FOS groups, but not in the HFD group: (D) Heatmap, (E) Ratio of rhythmic oscillation amplitude to *p*-value, (F) Ratio of rhythmic oscillation phase to amplitude. **G-J** Changes in the relative abundance of representative ASVs with rhythmic oscillations only in the LFD and HFD-FOS groups, with loss of oscillations in the HFD group (*n* = 6/group/time point): (G) ASV140, (H) ASV235, (I) ASV159, (J) ASV180. **K** Correlation analysis of representative ASVs with pro-inflammatory cytokines *Tnfα* and *Il6*. Oscillatory rhythms were detected by JTK_CYCLE algorithm in (G)-(J), * indicates *p* < 0.05.

The gut microbiota exhibits temporally dynamic patterns, with diurnal oscillations in taxonomic composition and species richness over 24-hour periods.^36^ We hypothesized that FOS regulates microbiota rhythmicity by reshaping microbial rhythms. Therefore, we first investigated whether FOS participates in regulating gut microbiota rhythms. We analyzed microbiota composition at six time points (08:00, 12:00, 16:00, 20:00, 00:00, and 04:00). PCoA revealed significant temporal differences in microbiota composition within the same group of mice (Figure 3A). Furthermore, although the overall temporal trend in total microbial reads was similar among the different groups of mice, the specific microbial taxa displaying rhythmicity varied (Figure 3B; Fig S7). We identified 21 ASVs exhibiting rhythmicity in both the LFD and HFD-FOS groups but not in the HFD group (Table S1; Figure 3C-F). Among them, ASV140 (Oscillospiraceae), ASV159 (*Parasutterella*), ASV180 (*Eisenbergiella*), ASV235 (Muribaculaceae), ASV211 (*NK4A214_group*), ASV175 (*GCA_900066575*), and ASV247 (Bacteroidia) showed significant inverse associations with *Tnfα* or *Il6* (Figure 3G-K). These findings suggest that FOS supplementation modulates gut microbiota rhythms and enhances the rhythmic expression of bacteria negatively associated with inflammation in HFD-fed mice.

### 3.4. Microbiota rhythmicity entrains colonic inflammatory oscillations

**Figure 4.**
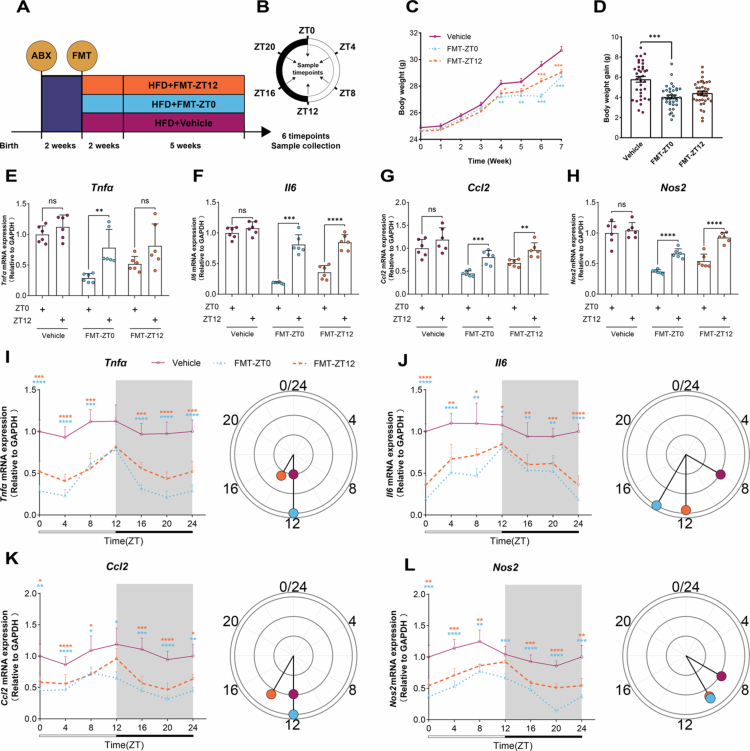
Microbiota rhythmicity entrains colonic inflammatory oscillations. **A-B** Schematic diagram of the experimental design: (A) Experimental design, (B) Assay time points. **C-D** Weight Change Chart: (C) Weight Change, (D) Weight Gain. **E-H** Diurnal variation of inflammatory markers (*n* = 6/group/time point): (E) *Tnfα*, (F) *Il6*, (G) *Ccl2*, (H) *Nos2*. **I-L** Rhythmic oscillations of inflammatory markers: Line plots show expression at different time points, the direction of the line in the circular plot indicates phase, and the line length represents amplitude (*n* = 6/group/time point): (I) *Tnfα*, (L) *Il6*, (J) *Ccl2*, (K) *Nos2*. One-way ANOVA was used to calculate *p* values in (C)-(D). NS, *p* > 0.05, *p < 0.05, **p < 0.01, ***p < 0.001. Two-tailed unpaired Welch’s t test was used to calculate *p* values in (E)-(H). NS, *p* > 0.05, **p < 0.01, ***p < 0.001, ****p < 0.0001. Brown-Forsythe and Welch ANOVA followed by Dunnett’s test were used to calculate *p* values in (I)-(L). *p < 0.05, **p < 0.01, ***p < 0.001, ****p < 0.0001.

The rhythmic oscillations of gut microbiota exhibit a close correlation with intestinal inflammation levels. To assess whether FOS-mediated modulation of diurnal variations in colonic inflammatory markers correlates with microbial rhythms, we transplanted donor-phase-specific fecal suspensions prepared from HFD-FOS group mice collected at ZT0 or ZT12 into two groups of mice fed HFD (Figure 4A-B). To further characterize the recipient phenotype, we additionally monitored body weight and related obesity-associated measurements, including weekly food intake and food efficiency ratio (Figure 4C-D; Fig S8). These data provided a more complete physiological context for interpreting the inflammatory effects of donor-phase-dependent FMT. The data demonstrated that both donor-phase-specific FMT groups restored diurnal variations in the mRNA levels of colonic inflammatory markers, including *Tnfα*, *Il6*, *Ccl2*, and *Nos2* (Figure 4E-H). However, the diurnal variation in *Tnfα* mRNA level was restored only in the FMT-ZT0 group (Figure 4E). Interestingly, the level of inflammation in the FMT-ZT0 group was lower than that in the FMT- ZT12 group, both in the overall analysis and at each individual time point (Fig S9; Figure 4I-L). Further analysis using the JTK_CYCLE algorithm revealed that the FMT- ZT0 group exhibited the highest amplitude in the rhythmic oscillations of these inflammatory markers, whereas the Vehicle group showed the lowest amplitude (Table S5; Figure 4I-L). These findings suggest that FOS improves diurnal variation in colonic inflammatory markers in HFD-fed mice by enhancing the amplitude of their rhythmic expression. Collectively, these findings demonstrate how microbial rhythmicity regulates chronic low-grade inflammation in the colon.

### 3.5. Glutathione metabolism emerges as the key rhythmic pathway linking microbial and host oscillations

**Figure 5.**
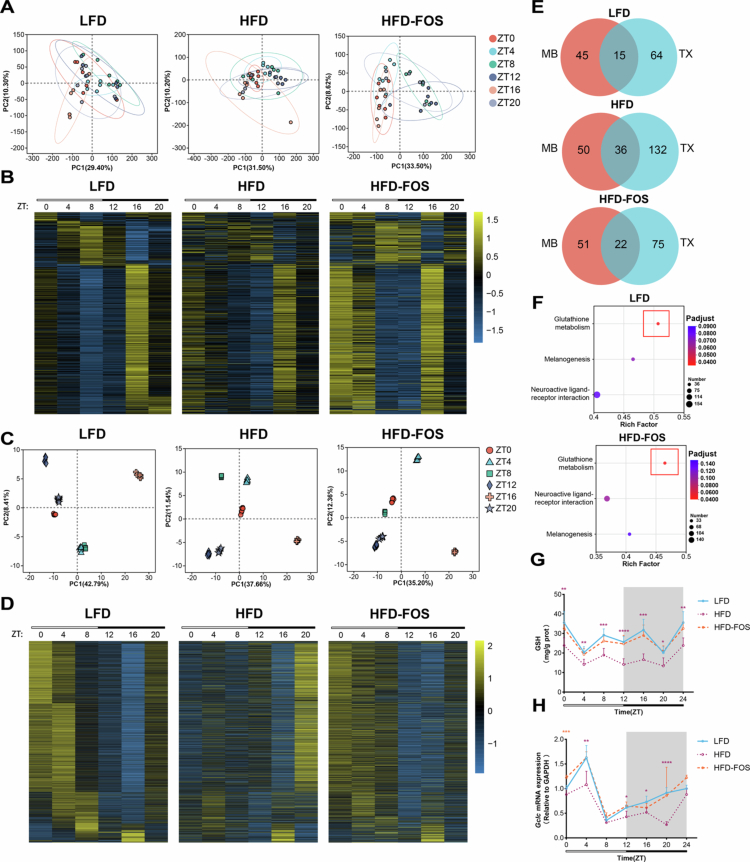
Glutathione metabolism emerges as the key rhythmic pathway linking microbial and host oscillations. **A** Comparison of metabolite *β*-diversity based on Bray-Curtis distance (*n* = 6/group/time point). **B** Metabolite rhythms analyzed using JTK_CYCLE algorithm, exemplified by heatmaps showing rhythms in both the LFD and HFD-FOS groups, and metabolites without rhythms in the HFD group. **C** Comparison of gene expression *β*-diversity (*n* = 6/group/time point). **D** Gene expression rhythms analyzed using JTK_CYCLE algorithm, reflected by heatmaps for genes with rhythms in both the LFD and HFD-FOS groups, and without rhythms in the HFD group. **E-F** Screening for pathways that overlap in the LFD and HFD-FOS groups but are not detected in the HFD group by analyzing the intersection of rhythmic metabolites with pathways enriched for rhythmic genes: (E) Venn diagram representing the intersection of metabolomics (MB) and transcriptomics (TX), (F) Enrichment bubble plots of pathways detected in both the LFD and HFD-FOS groups but not in the HFD group. **G** Line graph of rhythmic fluctuations in colonic glutathione content (*n* = 6/group/time point). **H** Line graph of rhythmic fluctuations in colonic *Gclc* gene expression (*n* = 6/group/time point). Brown-Forsythe and Welch ANOVA followed by Dunnett’s test were used to calculate *p* values in (G)-(H). *p < 0.05, **p < 0.01, ***p < 0.001, ****p < 0.0001.

We next aimed to elucidate how FOS regulates the rhythmic oscillations of colonic inflammatory markers via the gut microbiota. Given that gut microbiota-derived metabolites regulate host metabolism, immunity, and inflammation, we first collected colonic contents from three mouse groups at six time points and conducted metabolomic analyzes. JTK_CYCLE algorithm of the results revealed that fecal metabolite compositions exhibited dynamic rhythmic oscillations throughout the day, regardless of dietary conditions (Fig S10; Figure 5A). Subsequently, colon tissues were collected from three groups of mice at six time points for transcriptomic analyzes. JTK_CYCLE algorithm indicated that gene expression in the colon also exhibited temporal fluctuations (Fig S11; Figure 5C).

To explore rhythmic signaling pathways across the microbiota-host axis, we integrated JTK_CYCLE algorithm results with heatmaps depicting metabolites and genes exhibiting rhythmicity in both LFD and HFD-FOS groups, but not in the HFD group (Tables S2 and S3; Fig S10; Figure 5B and D). To further clarify the dynamic signaling pathways potentially restored by FOS—those normally present but disrupted by HFD—we performed a joint analysis of rhythmic metabolites and rhythmically expressed genes across all three dietary conditions. Potential signaling pathways were identified through KEGG pathway enrichment analysis (Figure 5E). The analysis revealed that 15 metabolic pathways, including the calcium signaling pathway, were enriched in the LFD group; 36 pathways, including those associated with insulin resistance, were enriched in the HFD group; and 22 pathways, with cGMP-PKG signaling as representative, were enriched in the HFD-FOS group (Table S4). Next, we identified three metabolic pathways shared by both the LFD and HFD-FOS groups but absent in the HFD group. Notably, among these pathways, the glutathione metabolism pathway showed the highest enrichment in both the LFD and HFD-FOS groups (Figure 5F), suggesting its potential role in mediating FOS-driven rhythmic oscillations in colonic inflammatory markers. Measurements of glutathione content across six time points and *Gclc* mRNA levels confirmed the significant regulatory effect of FOS on the colonic glutathione metabolic pathway (Figure 5G-H). In addition, oxidative stress-related indices, including colonic GSSG, MDA, and the GSH/GSSG ratio, further supported the presence of disturbed redox homeostasis in the HFD group, which was ameliorated by FOS treatment (Fig S12). Together, these findings suggest that the glutathione metabolism pathway may serve as a key link between microbial and host signaling in FOS-mediated modulation of circadian inflammatory oscillations in the colon.

### 3.6. Disruption of glutathione synthesis abolishes microbiota-driven restoration of inflammatory rhythms

**Figure 6.**
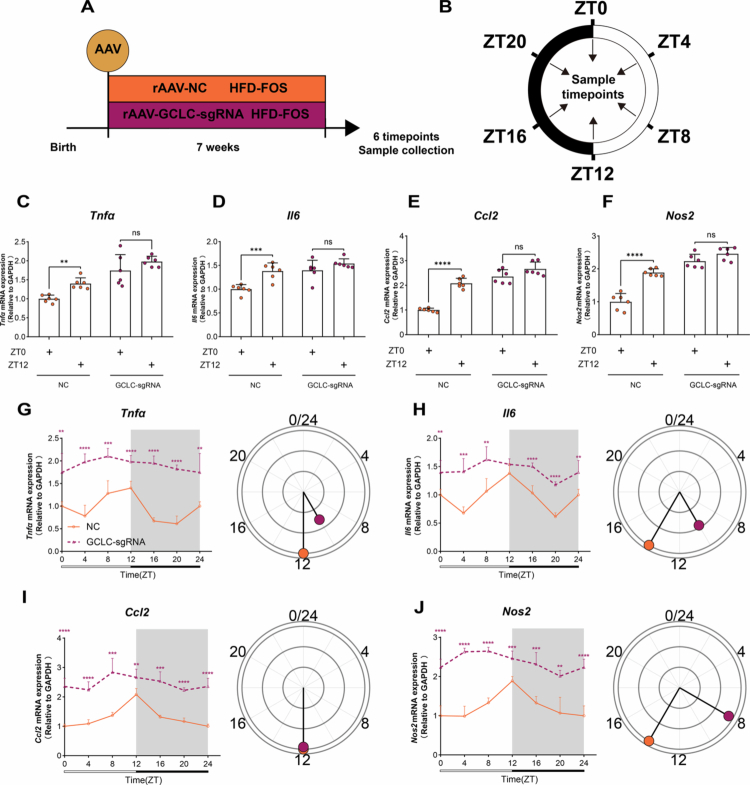
Disruption of glutathione synthesis abolishes microbiota-driven restoration of inflammatory rhythms. **A-B** Schematic diagram of experimental design: (A) Experimental design, (B) Detection time points. **C-F** Diurnal variation of inflammatory markers (*n* = 6/group/time point): (C) *Tnfα*, (D) *Il6*, (E) *Ccl2*, (F) *Nos2*. **G-J** Rhythmic oscillations of inflammatory markers: Line graphs show expression at different time points, the direction of the line in the circular graph indicates phase, and the line length represents amplitude (*n* = 6/group/time point): (G) *Tnfα*, (H) *Il6*, (I) *Ccl2*, (J) *Nos2*. Two-tailed unpaired Welch’s t test was used to calculate *p* values in (C)-(J). NS, *p* > 0.05, **p < 0.01, ***p < 0.001, ****p < 0.0001.

To elucidate the role of the glutathione metabolism pathway in the restoration of disrupted rhythmic oscillations of colonic inflammatory markers by FOS, we administered an adenoviral vector via enema to inhibit the expression of *Gclc* (glutamate-cysteine ligase catalytic subunit) in FOS-supplemented obese mice. (Figure 6A and B) Notably, suppression of *Gclc* expression abolished the ability of FOS to restore diurnal variation in colonic inflammatory markers, including *Tnfα*, *Il6*, *Ccl2*, and *Nos2*, in obese mice (Figure 6C-F). Furthermore, *Gclc* knockdown significantly elevated colonic inflammatory marker levels, both overall and at each individual time point (Fig S13). JTK_CYCLE algorithm further showed that inhibition of *Gclc* reduced the amplitude of rhythmic oscillations in inflammatory markers (Table S5; Figure 6G-J). These data indicate that glutathione metabolism is central to regulating rhythmic oscillations in colonic inflammatory markers.

### 3.7. Glutathione-dependent regulation of mitochondrial energy metabolism underlies restored colonic hypoxia rhythms

**Figure 7.**
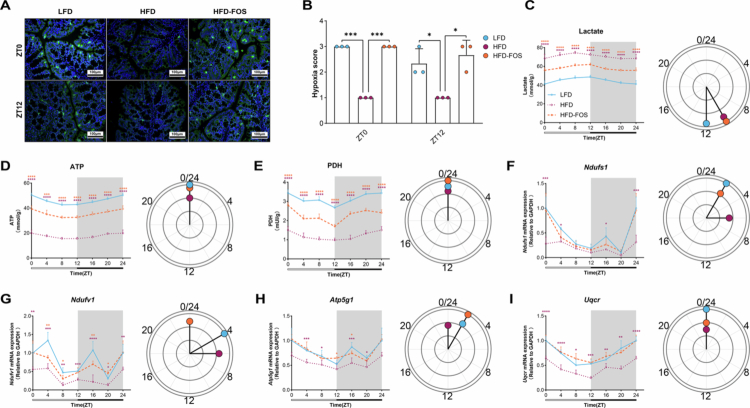
Glutathione-dependent regulation of mitochondrial energy metabolism underlies restored colonic hypoxia rhythms. **A-B** Hypoxia staining and scoring (*n* = 3/group/time point): (A) Hypoxia staining, (B) Hypoxia staining score. **C** Rhythmic oscillations in colonic lactate content (*n* = 6/group/time point). **D** Rhythmic oscillations in colonic ATP content (*n* = 6/group/time point). **E** Rhythmic oscillations in colonic PDH content (*n* = 6/group/time point). **F-I** Rhythmic expression of key genes in the mitochondrial respiratory chain (*n* = 6/group/time point): (F) *Ndufs1*, (G) *Ndufv1*, (H) *Atp5g1*, (I) *Uqcr*. One-way ANOVA was used to calculate *p* values in (B). *p < 0.05, ***p < 0.001 (theoretical limit due to complete separation). Brown-Forsythe and Welch ANOVA followed by Dunnett’s test were used to calculate *p* values in (C)-(I). *p < 0.05, **p < 0.01, ***p < 0.001, ****p < 0.0001.

Glutathione has been reported to influence redox homeostasis by modulating mitochondrial energy metabolism, thereby affecting inflammation levels.^37^^,^^38^ We first assessed colonic hypoxia using intraperitoneal injection of pimonidazole hydrochloride followed by immunostaining. The HFD-FOS group restored physiological colonic hypoxia compared to the HFD group (Figure 7A and B). To determine the involvement of colonic mitochondrial energy metabolism in modulating inflammatory marker oscillations, we measured colonic levels of lactate, ATP, and PDH. Data showed that, compared to the HFD group, ATP and PDH levels in the HFD-FOS group were elevated at all six time points, whereas lactate levels significantly decreased, accompanied by restored oscillation amplitudes (Figure 7C-E; Table S5). Analysis of mitochondrial respiratory chain function revealed significantly increased expression of key genes (*Ndufs1*, *Ndufv1*, *Atp5g1*, and *Uqcr*) at all six time points in the HFD-FOS group compared with the HFD group, along with enhanced rhythmic oscillation amplitudes (Table S5; Figure 7F-I). These results suggest that FOS restores rhythmic oscillations in mitochondrial electron transport chain activity in the colonic cells of obese mice, concurrently restoring physiological colonic hypoxia.

## 4. Discussion

The escalating global burden of metabolic syndrome, which is marked by obesity, insulin resistance, and cardiovascular complications, is increasingly attributed to chronic low-grade intestinal inflammation.^39-41^ The rhythmic oscillations of this inflammatory process are essential for gut homeostasis.^42^^,^^43^ Beyond elevating basal inflammatory tone, disruption of circadian inflammatory rhythms emerges as an additional pathological hallmark of metabolic disease, highlighting the importance of temporal regulation in immune-metabolic homeostasis.^7^^,^^44^ These rhythms function as active regulatory processes, arising from the dynamic interplay between host circadian clock genes and gut microbial oscillations.^14^^,^^16^ The host circadian system exerts top-down control of immune responses through cell-intrinsic transcriptional-translational feedback loops, whereas the gut microbiota displays autonomous diurnal fluctuations in composition and metabolic activity that shape host physiology via bottom-up signaling.^45^^,^^46^ Although microbial metabolites such as short-chain fatty acids and secondary bile acids have been recognized as intermediaries in microbiota-host circadian communication,^13^^,^^47^^,^^48^ the molecular mechanisms by which microbial rhythms convey temporal signals to regulate host inflammatory cycles during diet-induced metabolic disruption remain poorly understood. Here, we show that microbial rhythmicity regulates, at least in part, colonic inflammatory oscillations through a previously unrecognized microbiota-glutathione metabolic axis that operates largely independently of core clock genes. This finding supports a model in which the gut microbiota acts as a non-cell-autonomous regulator of host circadian physiology, thereby expanding the current view that circadian regulation is predominantly governed by the host clock. Identifying glutathione metabolism as a central pathway in microbiota-host communication represents an important conceptual advance, suggesting that glutathione metabolism may serve not only antioxidant functions^49^ but also a temporally regulated intermediary role in transmitting microbial signals to host inflammatory responses. These insights substantially broaden our understanding of how microbial communities shape host chronobiology and point to new mechanistic targets for therapeutic strategies aimed at resolving inflammation in metabolic disease.

Our initial observations revealed marked diurnal variations in colonic inflammatory markers under physiological conditions, which were completely abolished by HFD feeding. Notably, the lower inflammatory-factor levels observed at ZT0 relative to ZT12 likely reflect a physiological phase-dependent difference in mucosal inflammatory tone rather than a random fluctuation.^50^ Because mice are nocturnal animals, ZT12 corresponds to the onset of the active/dark phase, whereas ZT0 marks the onset of the light/rest phase. In this context, the higher inflammatory readouts at ZT12 may represent a normal time-of-day-dependent oscillatory state rather than a purely pathological increase.^51^ From this perspective, the key pathological effect of HFD is not simply that it elevates inflammatory markers, but that it disrupts their normal temporal organization, resulting in persistently elevated and arrhythmic inflammatory tone.^52^ This finding prompted a higher-resolution temporal analysis across six time points, which confirmed that HFD severely disrupted circadian rhythmicity,^53^ markedly dampening the amplitude of diurnal oscillations in key mediators, including *Tnfα*, *Il6*, *Ccl2*, and *Nos2*.^54^^,^^55^ This disruption led to persistently elevated inflammatory tone throughout the 24-hour cycle.^56^ Intervention with FOS effectively restored these rhythmic patterns via microbiota-dependent mechanisms, as antibiotic treatment completely abolished the effect. Strikingly, rhythmic restoration was also observed in intestine-specific *Bmal1* knockout mice (a core clock component),^57^ indicating that microbial signals can bypass host-intrinsic clock deficits and reestablish inflammatory rhythms through alternative pathways. The persistence of rhythmicity in clock-deficient mice under FOS treatment suggests that microbial-derived timing signals can functionally compensate for the loss of host transcriptional clock machinery, revealing an underappreciated redundancy in circadian regulatory networks. To understand how FOS mediates this compensatory effect, we next examined its impact on the gut microbiota itself. Temporal analysis of microbial community structure further revealed that FOS not only reshaped gut microbiota composition but also restored diurnal fluctuations of specific taxa, including *Marvinbryantia*, *ASF356*, and members of the *NK4A214_group*. Prior studies suggest that these taxa are biologically relevant in disease contexts including colitis-associated intestinal inflammation, vascular aging/endothelial dysfunction, metabolic dysfunction, or broader host inflammatory dysregulation, supporting their potential relevance to the present model.^33^^,^^34^^,^^58-60^ At the same time, direct evidence linking these specific taxa themselves to circadian rhythmicity remains limited, and therefore the rhythmic features emphasized here are derived primarily from our own data. In this context, the present findings suggest that FOS restores not only the abundance of selected inflammation-associated taxa, but also their temporal organization, underscoring that the timing of microbial signals is as critical as their identity in maintaining host inflammatory homeostasis. This conclusion was further supported by fecal microbiota transplantation experiments, which showed that donor fecal material collected at different circadian phases exerted distinct effects on host inflammatory rhythms, highlighting the functional importance of microbial temporal organization. Although the FMT experiment supports the transferability of donor-phase-dependent microbiota effects, it does not resolve which specific bacterial taxa or microbial products are responsible for the observed rhythmic effects.

Having established that microbial signals can restore inflammatory rhythms independently of the host clock, we next sought to delineate the underlying molecular mechanisms. We identified glutathione metabolism as a prominent rhythmic pathway linking microbial rhythmicity to host inflammatory oscillations.^61^ This interpretation is consistent with previous studies showing that glutathione levels, GCL activity, and Gclc-related biosynthetic programs exhibit circadian variation and are under clock-dependent regulation.^62^ Integrated multi-omics analyzes combining transcriptomic and metabolomic profiling revealed that glutathione metabolic oscillations were preserved in LFD and HFD-FOS groups but absent in HFD-fed mice, with the glutamate-cysteine ligase catalytic subunit (*Gclc*) displaying particularly robust circadian expression patterns that correlated with microbial diurnal dynamics.^63^^,^^64^ These oscillations encompassed both glutathione synthesis and regeneration, forming a coherent metabolic rhythm aligned with microbial ecological shifts.^65^ Functional validation through colon-specific *Gclc* knockdown showed that glutathione synthesis is functionally required for maintaining inflammatory rhythms, as its inhibition markedly dampened rhythmic amplitude and abolished the restorative effects of microbial rhythmicity. Consistent with this interpretation, the altered colonic glutathione homeostasis in the HFD group was also accompanied by increased GSSG and MDA levels together with a reduced GSH/GSSG ratio, indicating disturbed redox homeostasis. Thus, the reduction in colonic GSH in our model is most appropriately interpreted as reflecting both impaired glutathione synthesis and increased oxidative demand, rather than biosynthetic impairment alone. Mechanistically, the glutathione axis operates through redox-sensitive pathways that modulate inflammatory tone, potentially via regulation of reactive oxygen species (ROS), thereby creating a permissive environment for circadian immune control.^66^^,^^67^ Collectively, these findings support a temporally regulated role for glutathione metabolism in linking microbial rhythmicity to intestinal inflammatory homeostasis, consistent with its function as a chronometabolic interface between microbial ecology and host physiological timing. This interpretation is supported by convergent evidence from pathway enrichment, rhythmic changes in colonic glutathione content, oscillatory *Gclc* expression, altered redox homeostasis, and the loss-of-function effect of colon-specific *Gclc* knockdown.

The glutathione pathway serves as an important interface between microbial ecology and host redox homeostasis, orchestrating multi-level regulation of intestinal metabolic and inflammatory processes.^68^^,^^69^ Its rhythmic activity was linked to broad improvements in mitochondrial function, including enhanced ATP production,^70^ increased PDH activity,^71^ more efficient electron transport chain performance,^72^ and reduced lactate accumulation,^73^ which collectively indicate a metabolic shift toward oxidative phosphorylation. These functional enhancements were accompanied by elevated transcriptional amplitude of key mitochondrial respiratory chain genes (*Ndufs1*, *Ndufv1*, *Atp5g1*, and *Uqcr*),^74-77^ as determined by multi-timepoint analysis. This demonstrates a robust restoration of circadian rhythmicity and suggests a time-dependent optimization of energy metabolism under glutathione-mediated regulation.^78^ Furthermore, glutathione-associated restoration of mitochondrial rhythms was coupled with normalization of colonic oxygen homeostasis.^79^ Pimonidazole-based hypoxyprobe staining demonstrated that HFD feeding disrupted the physiological hypoxia characteristic of a healthy colon,^80^ yielding significantly lower hypoxia scores compared to the LFD group. Importantly, FOS intervention restored hypoxia to physiological levels, as indicated by higher hypoxia scores relative to the HFD group, thereby recovering the normal hypoxic microenvironment. This re-establishment of physiological hypoxia is critical for intestinal homeostasis, as it likely stabilizes the mucosal environment by promoting oxygen-sensitive beneficial commensals while constraining oxygen-tolerant pathobionts.^81^^,^^82^ Together, the coordinated recovery of redox balance, mitochondrial bioenergetics, and tissue hypoxia demonstrates how microbial-driven glutathione oscillations synchronize cellular metabolism with environmental conditions to sustain intestinal health.

In conclusion, our findings support a model in which microbial rhythms are transduced through glutathione-dependent mechanisms to entrain host inflammatory and metabolic cycles. This microbiota-glutathione-mitochondria axis constitutes an additional layer of temporal regulation that functions in parallel with the canonical clock network,^83^^,^^84^ thereby maintaining circadian homeostasis even when core clock components are impaired.^85^ The ability of this microbial-metabolic circuit to operate independently of host transcriptional oscillators suggests an evolutionarily conserved complementary mechanism that supports temporal regulation of intestinal functions under diverse physiological conditions. These insights broaden our understanding of how microbial communities influence host chronobiology,^86^^,^^87^ and support the view that microbes may act as active participants in circadian timekeeping rather than merely passive responders to host rhythms. Moreover, this work highlights new mechanistic targets for chronotherapeutic strategies aimed at mitigating inflammation in metabolic disease. Future studies focused on targeted manipulation of the glutathione cycle or on specific microbial producers of glutathione-regulating metabolites may yield novel approaches for correcting circadian disruptions associated with obesity and related disorders. Finally, the discovery that a fundamental cellular antioxidant system serves as a temporal interface between microbes and host physiology opens new avenues for elucidating how metabolic pathways integrate environmental and biological time cues to orchestrate physiological homeostasis and combat disease.

## 5. Limitations of the study

While our study identifies the microbiota-glutathione axis as a key regulator of colonic inflammatory rhythms, several limitations warrant consideration. First, the specific microbial strains and species driving rhythmic glutathione metabolism remain unidentified. Identifying these chronobiotic microbes beyond community-level analysis will require integrated approaches that combine gnotobiotic models with strain-resolved metabolomics. Second, although we observed spatiotemporal coupling between luminal and tissue glutathione oscillations, the mechanistic link—namely, how rhythmic microbial metabolites signal to epigenetically or transcriptionally regulate host epithelial redox genes—remains unclear. Spatial multi-omics and metabolic flux analyzes may help elucidate this cross-compartmental dialog. Finally, to evaluate the broader translational impact of our findings, future studies should: (1) determine whether the identified rhythmic microbial-metabolic axis represents a general mechanism that can be entrained by prebiotics or dietary interventions beyond FOS; and (2) investigate the therapeutic potential of targeting this axis in other circadian-disrupted inflammatory conditions, such as post-infectious IBS or IBD.

## Supplementary Material

Table S1.xlsx Table S1.xlsx

Table S8.xlsx Table S8.xlsx

Table S7.docx Table S7.docx

Supplementary Figures.docx Supplementary Figures.docx

Table S2.xlsx Table S2.xlsx

Table S4.xlsx Table S4.xlsx

Table S5.xlsx Table S5.xlsx

Table S3.xlsx Table S3.xlsx

Table S6.docx Table S6.docx

## Data Availability

All sequencing and omics datasets generated in this study are publicly available. The 16S rRNA sequencing data are available at NCBI BioProject (PRJNA1419007). The transcriptome and untargeted metabolomics data are available at CNCB (NGDC) via GSA and OMIX (Project: PRJCA057885). All raw data for the main figures and supplementary materials are available on Figshare (https://doi.org/10.6084/m9.figshare.32054556).
