# Supplementary material for: Gut microbiota-regulated glutathione metabolic rhythms restore obesity-induced colonic inflammatory oscillations
Source: Gut Microbes. 2026 May 9;18(1):2670048. doi: 10.1080/19490976.2026.2670048 (PMC13166208; doi:10.1080/19490976.2026.2670048)
Supplement: Table S7.docx [file KGMI_A_2670048_SM6022.docx]

Table S7. Histopathological evaluation criteria for intestinal inflammation severity and epithelial surface integrity

| Histologic feature | Score | Criteria |
| --- | --- | --- |
| Infiltration by polynuclear cells | 0 | rare neutrophils within lamina propria |
|  | 1 | occasional multifocal increased frequency  of neutrophils within lamina propria |
|  | 2 | diffuse infiltration of the lamina propria by  low numbers of neutrophils |
|  | 3 | diffuse infiltration of the submucosa and  lamina propria by moderate numbers of  neutrophils |
|  | 4 | diffuse transmural infiltration of the  submucosa, lamina propria, and muscle  layers by high numbers of neutrophils with  accumulation of luminal suppurative  exudate |
| Infiltration by mononuclear cells | 0 | no detectable increase in mononuclear  infiltration of the lamina propria |
|  | 1 | mild multifocal expansion of the lamina  propria by low numbers of infiltrating  lymphocytes and plasma cells |
|  | 2 | mild regional expansion of the lamina  propria by moderate numbers of  lymphocytes and plasma cells |
|  | 3 | moderate regional to diffuse expansion of  the lamina propria and epithelia by  infiltrating lymphocytes and plasma cells |
|  | 4 | marked diffuse expansion of the lamina  propria and epithelia by infiltrating  lymphocytes and plasma cells with  distortion of the mucosal architecture |
| Submucosal edema | 0 | no observable edema |
|  | 1 | mild multifocal perivascular expansion of  the tunica adventitia |
|  | 2 | moderate multifocal expansion of the  submucosa with lymphatic dilation |
|  | 3 | severe circumferential expansion of the  submucosa with marked lymphatic dilation |
| Luminal exudate accumulation | 0 | no observable accumulation of  suppurative exudate within the lumen |
|  | 1 | mild accumulation of exudate |
|  | 2 | moderate accumulation exudate |
|  | 3 | severe accumulation of exudate |
| Superficial epithelial injury | 0 | no observable disruption to the brush  border |
|  | 1 | occasional multifocal brush border  attenuation with increased apoptosis |
|  | 2 | frequent to confluent brush border  attenuation with abundant apoptosis |
|  | 3 | marked brush border attenuation with loss  of epithelial adhesion |
|  | 4 | surface epithelial cell loss leading to erosive lesions of the superficial mucosa |
|  | 5 | frequent erosive lesions with confluence leading to ulceration |
