# Supplementary material for: Gut microbiota-regulated glutathione metabolic rhythms restore obesity-induced colonic inflammatory oscillations
Source: Gut Microbes. 2026 May 9;18(1):2670048. doi: 10.1080/19490976.2026.2670048 (PMC13166208; doi:10.1080/19490976.2026.2670048)
Supplement: Supplementary Figures.docx [file KGMI_A_2670048_SM6023.docx]

**Supplementary Figures**

**Gut Microbiota-Regulated Glutathione Metabolic Rhythms Restore Obesity-Induced Colonic Inflammatory Oscillations**

**Zhenting Zhao^1^, Renjie Shi^1^, Jin Ye^1^, Danna Wang^1^, Beita Zhao^1^, Bo Ren^2^, Luanfeng Wang^3^, Xiaoning Liu^4^, Xuebo Liu^1*^**


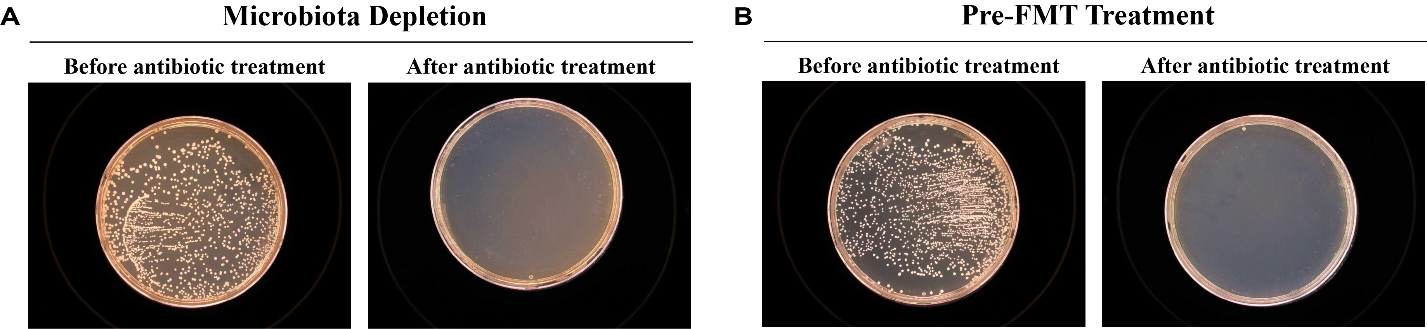
**Fig S1.** Representative images of BHI agar plates.

**A** Differences before and after antibiotic treatment in the microbiota-dependent experiment.


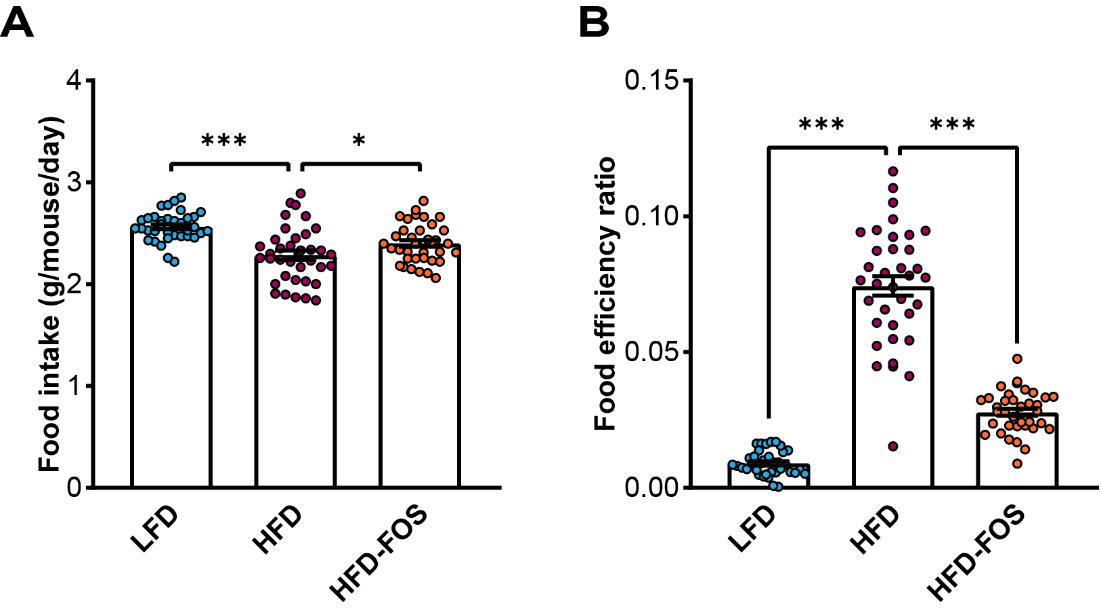
**B** Differences before and after antibiotic treatment during pre-treatment prior to FMT.

**Fig S2.** Food intake and feed efficiency ratio.

**A** Food intake.

**B** Feed efficiency ratio.

**One-way ANOVA was used to calculate *p* values in (A) and (B). ***p* < 0.01, ****p* < 0.001.**


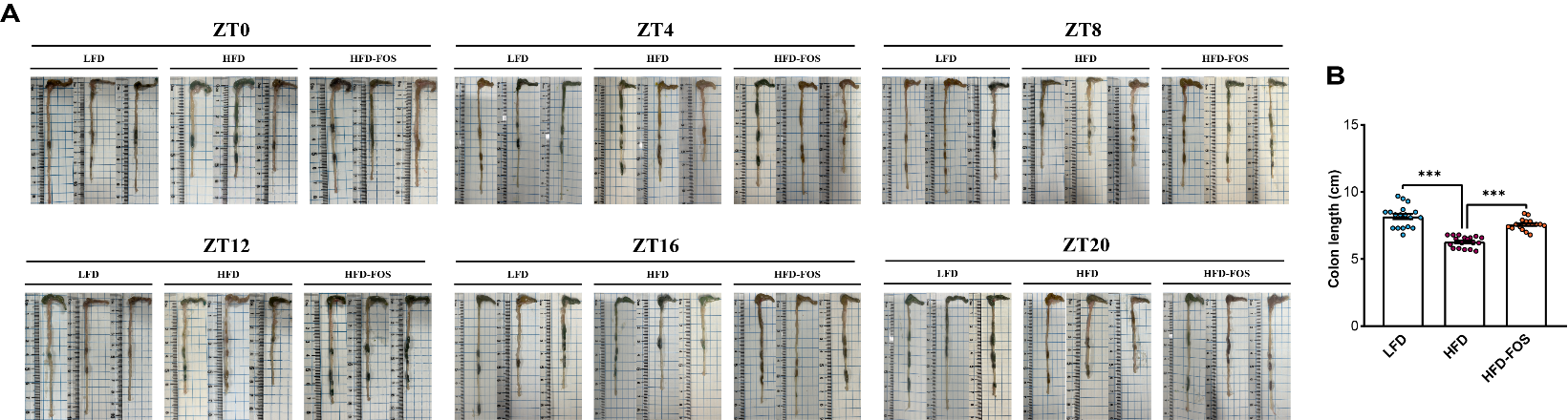
**Fig S3.** Colon images at six time points and differences in their lengths.

**A** Colon images at six time points.

**B** Differences in colon length.

**One-way ANOVA was used to calculate *p* values in (B). ****p* < 0.001.**


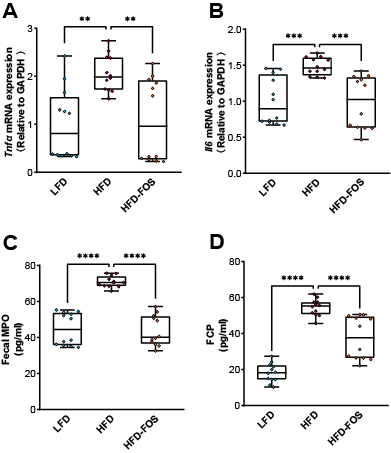
**Fig S4.** FOS improves colonic inflammatory marker levels in obese mice.

**A–D** Colonic inflammatory marker levels (n=12/group): (A) *Tnfα*, (B) *Il6*, (C) MPO, (D) FCP.


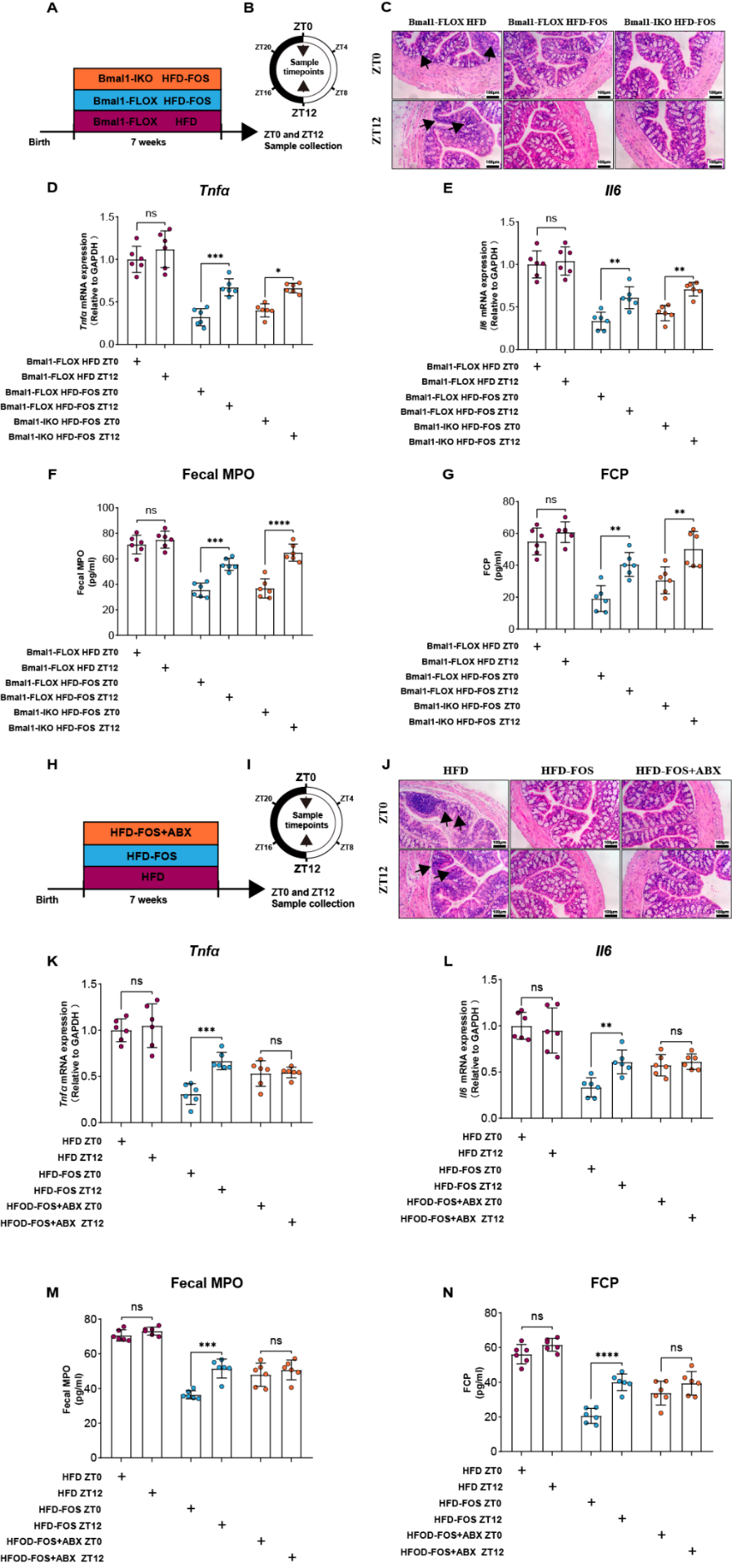
**One-way ANOVA was used to calculate *p* values in (A)–(D). ***p* < 0.01, ****p* < 0.001, **p* < 0.0001.**

**Fig S5.** FOS requires the gut microbiota but not the intestinal circadian clock to restore diurnal variations in inflammatory markers.

**A–B** Schematic diagram of the experimental design: (A) Experimental design, (B) Assay time points.

**C** H&E staining (n=3/group/time point).

**D–G** Diurnal variations in colonic inflammatory markers in mice (n=6/group/time point): (D) *Tnfα*, (E) *Il6*, (F) Fecal MPO, (G) Fecal FCP.

**H–I** Schematic diagram of the experimental design: (H) Experimental design, (I) Detection time points.

**J** H&E staining.

**K–N** Diurnal variations in colonic inflammatory markers in mice: (K) *Tnfα*, (L) *Il6*, (M) Fecal MPO, (N) Fecal FCP.

**Two-tailed unpaired Welch’s t test was used to calculate *p* values in (D)–(G) and (K)–(N). NS, *p* > 0.05, **p* < 0.05, ***p* < 0.01, ****p* < 0.001, *****p* < 0.0001.**


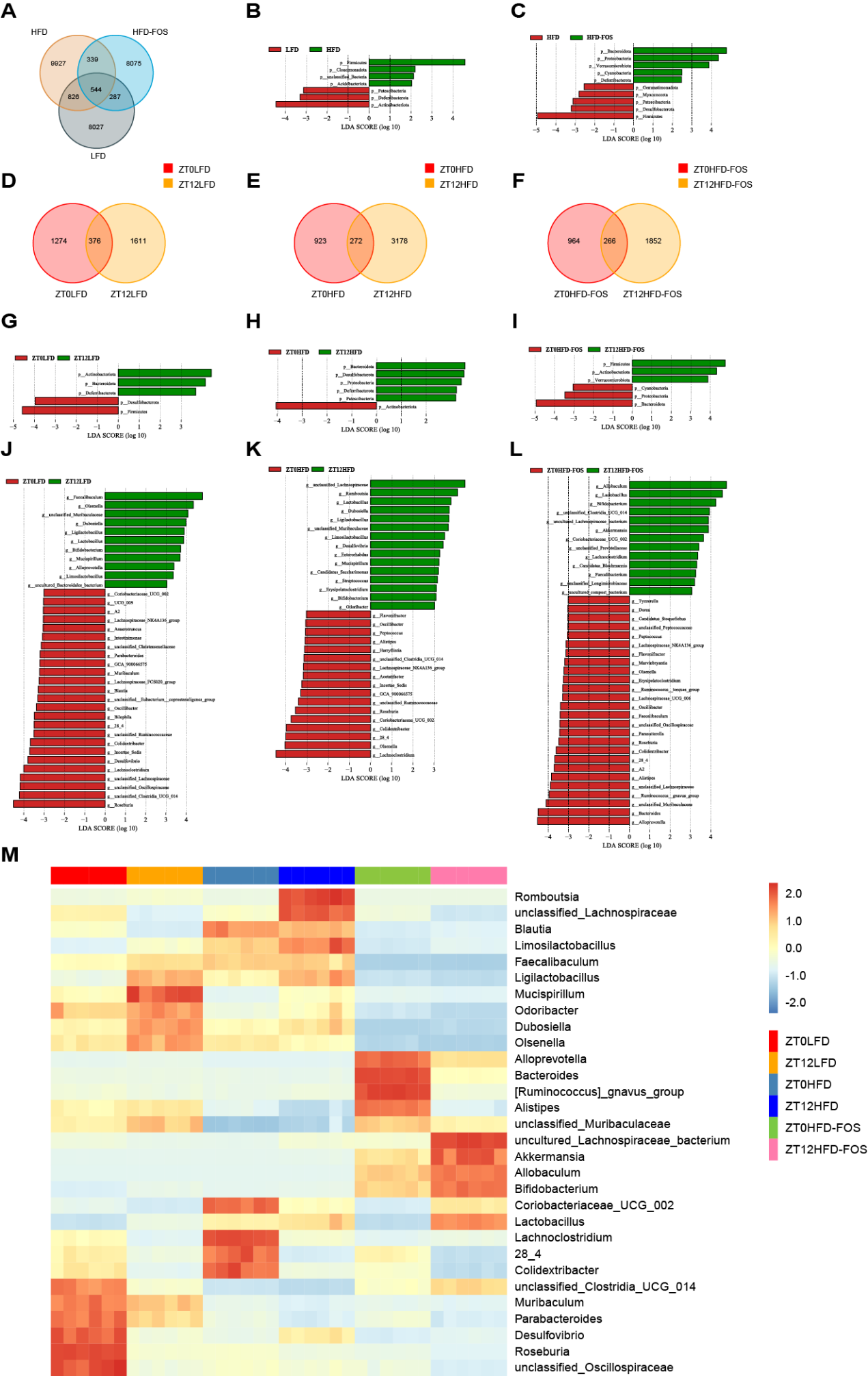


**Fig S6.** Diet and time collectively shape gut microbiota composition.

**A** Venn diagrams of gut microbiota composition under different diets.

**B–C** Comparison of differences in genus levels under different diets: (B) LFD vs. HFD, (C) HFD vs. HFD-FOS.

**D–F** Comparison of ZT0 and ZT12 Venn diagrams: (D) LFD, (E) HFD, (F) HFD-FOS.

**G–I** Comparison of phylum-level differences between ZT0 and ZT12: (G) LFD, (H) HFD, (I) HFD-FOS.

**J–L** Comparison of genus-level differences between ZT0 and ZT12: (J) LFD, (K) HFD, (L) HFD-FOS.

**M** Heatmap of genus-level differences.


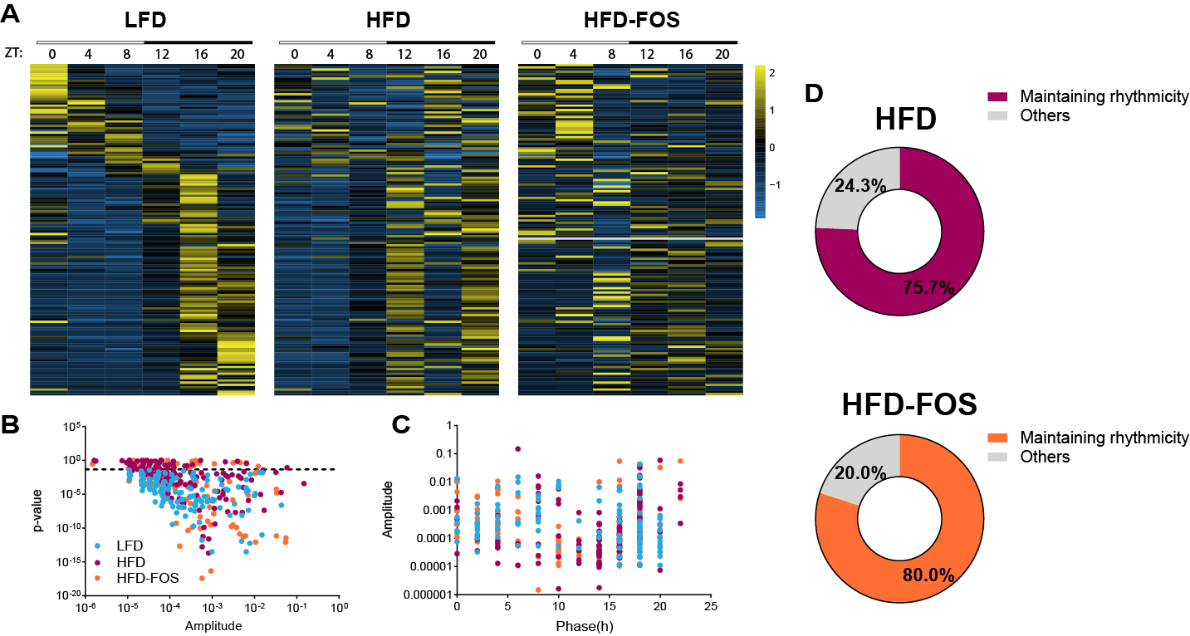
**Fig S7.** FOS modulates gut microbiota rhythmic oscillations.

**A–C** Oscillation patterns of rhythmic ASVs in the LFD group under various dietary conditions: (A) Heatmap, (B) Ratio of rhythmic oscillation amplitude to p-value, (C) Ratio of rhythmic oscillation phase to amplitude.

**D** Percentage of relative abundance of rhythmic ASVs in the LFD group that remained rhythmic in other groups.


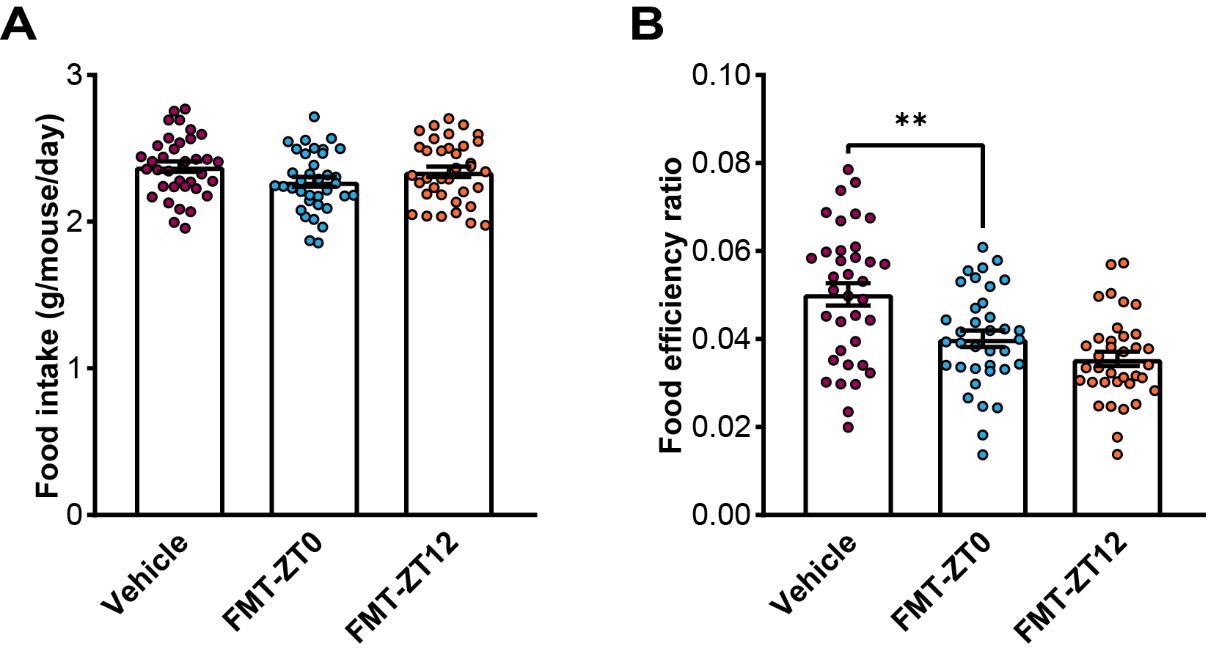
**Fig S8.** Food intake and feed efficiency ratio.

**A** Food intake.

**B** Feed efficiency ratio.

**One-way ANOVA was used to calculate *p* values in (A) and (B). ***p* < 0.01.**


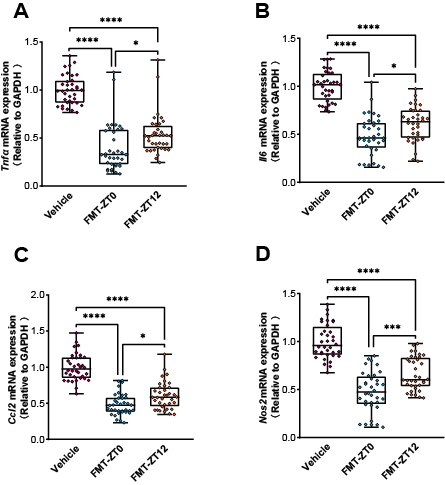
**Fig S9.** Fecal bacterial transplantation (FMT) improves colonic inflammatory marker levels in obese mice.

**A–D** Colonic inflammatory marker levels (n=36/group): (A) *Tnfα,* (B) *Il6*, (C) *Ccl2*, (D) *Nos2*.

**One-way ANOVA was used to calculate p values in (A)–(D).** ****p* < 0.05, ****p* < 0.001, *****p* < 0.0001.**


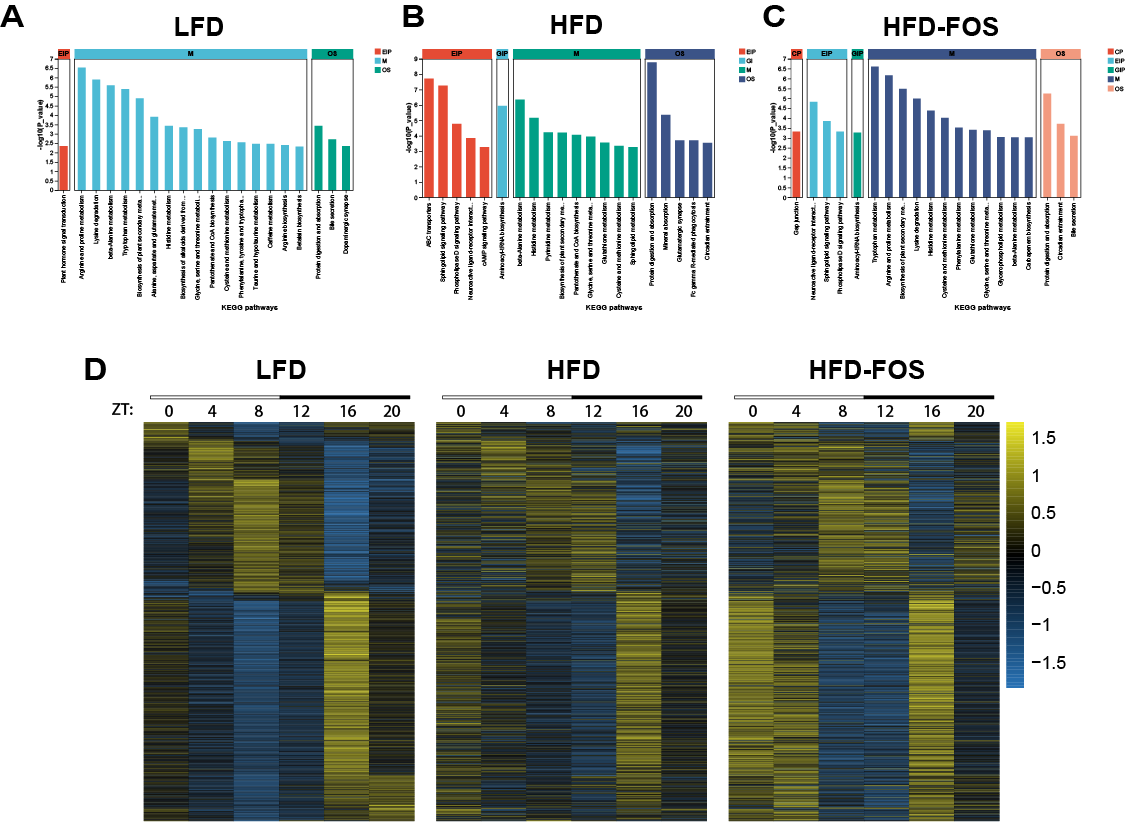
**Fig S10.** FOS modulates rhythmic oscillations of metabolites in colonic contents.

**A–C** Rhythmic metabolites were screened by JTK_CYCLE algorithm for different diets and enriched for KEGG pathways: (A) LFD, (B) HFD, (C) HFD-FOS. CP: Cellular Processes; EIP: Environmental Information Processing; GIP: Genetic Information Processing; M: Metabolism; OS: Organismal Systems.

**D** Heatmap of oscillation patterns of rhythmic metabolites in the LFD group under various dietary conditions.


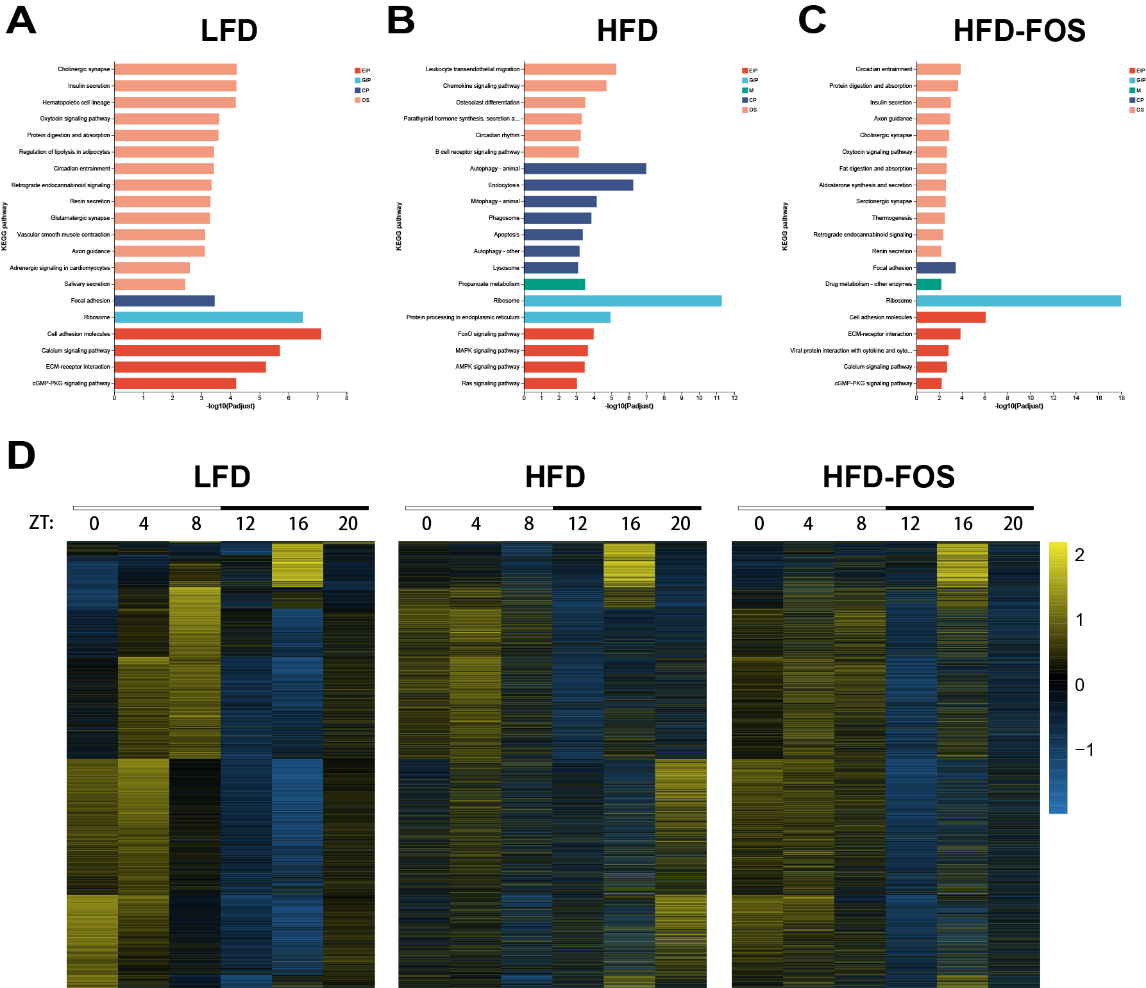
**Fig S11.** FOS regulates rhythmic oscillations of genes in colonic tissues.

**A–C** Rhythmically expressed genes were screened by JTK_CYCLE algorithm for different diets and enriched for KEGG pathways: (A) LFD, (B) HFD, (C) HFD-FOS. CP: Cellular Processes; EIP: Environmental Information Processing; GIP: Genetic Information Processing; M: Metabolism; OS: Organismal Systems.


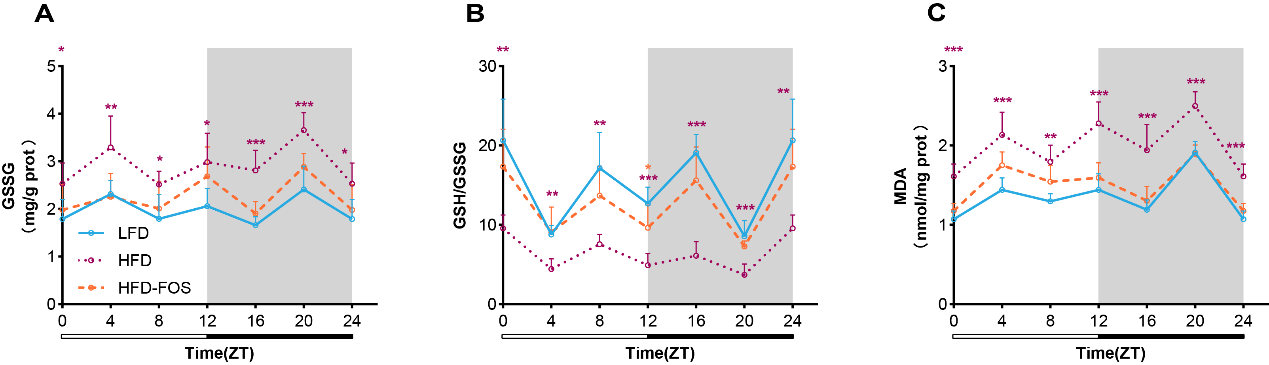
**D** Heatmap of oscillation patterns of rhythmic genes in the LFD group under various dietary conditions.

**Fig S12.** Oxidative stress-related markers.

**A** Colon GSSG content.

**B** Colon GSH/GSSG ratio.

**C** Colon MDA content.

**D** Heatmap of oscillation patterns of rhythmic genes in the LFD group under various dietary conditions.

Brown-Forsythe and Welch ANOVA followed by Dunnett’s test were used to **calculate *p* values in (A)–(C). **p* < 0.05, ***p* < 0.01, ****p* < 0.001, *****p* < 0.0001.**


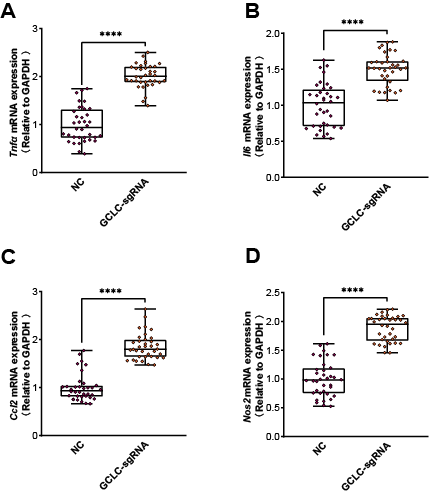
**Fig S13.** Inhibition of the colonic glutamate–cysteine ligase catalytic subunit (*Gclc*) gene suppresses the ameliorative effect of FOS on colonic inflammatory markers in obese mice

**A–D** Levels of colonic inflammatory markers (n=36/group). (A) *Tnfα,* (B) *Il6*, (C) *Ccl2*, (D) *Nos2*.

**Two-tailed unpaired Welch’s t test was used to calculate *p* values in (A)–(D). *****p* < 0.0001.**
