# Supplementary material for: Gut microbiota-regulated glutathione metabolic rhythms restore obesity-induced colonic inflammatory oscillations
Source: Gut Microbes. 2026 May 9;18(1):2670048. doi: 10.1080/19490976.2026.2670048 (PMC13166208; doi:10.1080/19490976.2026.2670048)
Supplement: Table S6.docx [file KGMI_A_2670048_SM6030.docx]

|  | Forward sequence (5’ – 3’) | Reverse sequence (5’ – 3’) |
| --- | --- | --- |
| *Gclc* | AGACACGGCATCCTCCAGTT | CTGACACGTAGCCTCGGTAA |
| *Tnfα* | AGCCAGGAGGGAGAACAGAAAC | CCAGTGAGTGAAAGGGACAGAACC |
| *Ccl2* | AGGTGTCCCAAAGAAGCTGTA | ATGTCTGGACCCATTCCTTCT |
| *Il6* | TAGTCCTTCCTACCCCAATTTCC | TTGGTCCTTAGCCACTCCTTC |
| *Nos2* | TTGGGTCTTGTTCACTCCACGG | CCTCTTTCAGGTCACTTTGGTAGG |
| *Ndufs1* | AGCCGGCAGCCATCATGTTA | AGTTACTTGCTGCTGTGCCA |
| *Ndufv1* | TTTCTCGGCGGGTTGGTTC | GGTTGGTAAAGATCCGGTCTTC |
| *Uqcr* | GGACTGGGTGCCTTACATCAACG | GTGTCCAGCTTCCTCAGTGTCTTC |
| *Atp5g1* | TTCTCCAGCTCTGATTCGCTC | CCGGGAAATGACACTGGTCT |
| *Gapdh* | TGGAGAAACCTGCCAAGTATGA | TGGAAGAATGGGAGTTGCTGT |

Table S6. Primers for real-time PCR of mouse genes
